# Regression tree modelling to predict total average extra costs in household spending during COVID-19 pandemic

**DOI:** 10.1186/s42269-021-00585-z

**Published:** 2021-07-19

**Authors:** Nesma Lotfy

**Affiliations:** Department of Biostatistics, High Institute of Public Health, Alexandria University, Alexandria, Egypt

**Keywords:** Household spending, COVID-19, Costs, Regression tree

## Abstract

**Background:**

Prevention of coronavirus (COVID-19) regarding households has many aspects, such as buying mask, hand sanitizer, face shield, and many others. As a result of buying the previous items, the household spending per month will increase during the COVID-19 pandemic period. This study aimed to calculate the average costs of each extra item involved in households spending during COVID-19 pandemic and to predict the total average extra costs spending by households.

**Results:**

Most of the respondents were females (81%) and aged between 30 and 40 (56.3%). About 63.1% of families had the same monthly income while 35.4% had a decrease in monthly income. A significant reduction in days of leaving home before and after COVID-19 pandemic was observed (before; median = 6, after; median = 5, *P* =  < 0.001). The extra spending in grocery was the dominated item compared to other items (mean = 707.2 L.E./month, SD = 530.7). Regarding regression tree, the maximum average extra costs due to COVID-19 pandemic were 1386 L.E./month (around 88.56$/month (1$—> 15.65L.E.)) while the minimum average extra costs were 217 L.E./month (around 13.86$/month).

**Conclusions:**

The effect of COVID-19 pandemic in households spending varies largely between households, it depends on what they do to prevent COVID-19.

## Background

The coronavirus disease-2019 (COVID-19) is an ongoing worldwide pandemic caused by severe acute respiratory syndrome coronavirus 2 (SARS CoV-2). The virus started in Wuhan, China, but then it spread to all over the world. On January 2020, the World Health Organization declared that the COVID-19 outbreak as a global health emergency. Later, it declared it as a global pandemic (WHO 2020a).

The COVID-19 virus infects all ages. However, older-aged people and patients with underlying medical conditions (such as hypertension, diabetes, cardiovascular disease, chronic respiratory disease, chronic kidney disease, immunocompromised status, cancer, pregnancy, smoking, and obesity) are at a higher risk of severe COVID-19 disease. Severe COVID-19 disease causes hospitalization, admission to the ICU, intubation or mechanical ventilation, or death (Garg et al. 2020).

Prevention of coronavirus (COVID-19) is through regular hand washing and covering mouth and nose during sneezing or coughing. Avoidance of closed spaces and crowded places is also crucial for prevention of viral spread (WHO 2020a, b).

From our daily life in Alexandria, Egypt, there are also many undertaken promotions to prevent COVID-19 such as taking dietary supplements (vitamin C, Zinc, etc.), conduct periodic examinations, use car transportation service such as Uber instead of public transportation, increase the school expenses due to COVID-19, and many others. Thus, the main objective of the current study is to calculate the average extra costs of each item involved in households spending and to build a model to predict the total average extra costs spending by households due to COVID-19 pandemic.

## Methods

### Study design

A cross-sectional study was conducted from October 2020 to November 2020 at High Institute of Public Health (HIPH), Alexandria University.

### Sampling design

Exponential snowball sampling technique was done to recruit HIPH students, their friends, and families.

### Data collection methods and tools

A predesigned structured questionnaire was created to measure the impact of COVID-19 situation on the economics of Egyptian households. Content validity was assessed by three professors at HIPH, they agreed about the main items that must be asked to measure the average extra costs due to COVID-19 pandemic which are masks, face shields, treatment of COVID-19, hand wash, chlorine, hand sanitizers, vitamins, periodic examination, transportation, delivery service, grocery spending, school expenses, and university expenses.

### Data management

The questionnaire was completed by a total of 280 respondents, about 17 (6.1%) individuals submitted more than one application so the duplicates were excluded from the study, and the remaining were 263 respondents. The data was collected online using google form.

### Data cleaning

After collection of data, it was coded and fed to SPSS (statistical package for the social sciences) (IBM Corp. 2017).

The costs were trimmed to remove the extreme low and extreme high values, so only values between 10% percentile and 90% percentile were entered in calculation.

- Calculation of average extra costs for each item per month
  - Average cost of masks per month if they were bought.
  - Average cost of face shields for children if they were bought.
    - It was assumed that the face shields will be bought every 3 months), so the average cost of face shields per month was calculated as follows:
        (Cost of face shields * number of face shields bought)/3
  - Average cost of treatment for COVID-19 if someone was infected in the family.
    - It was assumed that the individual that was infected will be infected only one time per year, so the average cost of treatment per month was calculated as follows:
        (Cost of treatment * number of infected individuals)/12
  - Average extra cost of hand wash per month.
  - Average extra cost of chlorine per month.
  - Average cost of hand sanitizers if they were bought.
    - It was calculated as follows:
        Cost of hand sanitizers * number of bottles used per month.
  - Average costs of taking vitamins per month if they were bought.
  - Average costs of mouth gargle bottles if they were bought.
    - It was assumed that 2 bottles will be used per month, so average costs of mouth gargle per month was calculated as follows:
        Cost of one bottle * 2
  - Average cost of periodic examination per month.
  - Average extra cost of using Uber or Taxi instead of public transportation if they use.
    - Increasing cost calculated as: the cost of using Uber or Taxi per days for one person in the family subtracting by the cost of public transportation (around 15 L.E. per day)
    - The increasing cost was multiplied by 15 days (average days of coming out from house)
  - Average extra cost of using delivery service if they use.
    - If they were usually use delivery service before COVID-19 situation and then stops, it was calculated as follows:
      - (Delivery service fees (max 20 L.E.)) * 15 days
    - If they were usually not use delivery service before COVID-19 situation and then use it.
        (Delivery service fees (max 20 L.E.)) * 15 days
  - Average extra cost of grocery spending per month.
  - Average extra costs of school expenses for children per month.
    - It was calculated as follows:
        Extra costs for all children/12
  - Average extra costs of university expenses for students per month.
    - It was calculated as follows:
        (Extra costs for one student * number of students)/12
- Calculation of total average extra cost
  - Total cost was calculated as follows:
      First, calculate the average extra cost for each item according to the whole responding population. This was done by multiplying each item cost with the proportion of responding population who paid extra cost for it. For example, if individual_1 paid for masks 100 L.E., and 95% of responding population uses masks, then the cost for this individual will be 100*0.95 = 95 L.E.
      Second, sum all average extra costs for all items, to get the total extra costs for each individual.

### Statistical analysis

#### Descriptive analysis

Descriptive statistics were calculated using min, max, mean, and standard deviation (SD) for the numeric data and frequency, and percent for categorical data.

Wilcoxon sign rank test was conducted to test the significant difference between household leaving days before and after COVID-19 pandemic. Moreover, median, first quartile (Q1), and third quartile (Q3) were also represented.

#### Regression tree modelling

Regression tree modelling technique using CART (Classification and Regression Trees) algorithm (Breiman et al. 1984) was implemented to build a prediction model in terms of if-else rules to predict the total average extra costs spend per month during COVID-19 period based on its items. The regression tree has root nodes and leaf nodes. The root nodes represent a single input value (x) and a split point on that variable, while the leaf nodes represent the output variable (y). The CART algorithm involving selecting the input variables is based on a greedy algorithm which depends on minimization of sum square errors. The analysis was carried out using the R Statistical Environment (R Core Team 2017) through “rpart” package (Therneau and Atkinson 2019).

## Results

Concerning to characteristics of respondents, more than half of respondents age between 30 and 40 (56.3%), females (81%), married (62.4%), and from Alexandria (71.5%) (Table 1).

**Table 1:** Demographic characteristics of the respondents

Variable	N (%) N = 263
Age	
21–	67 (25.5)
30–	148 (56.3)
40 +	48 (18.2)
Gender	
Male	50 (19.0)
Female	213 (81.0)
Marital status	
Married	164 (62.3)
Single and live with parents	74 (28.1)
Single (live alone)	8 (3.0)
Widow or divorce	17 (6.5)
Number of individuals in a family	
1	13 (4.9)
2	21 (8.0)
3	57 (21.7)
4	90 (34.2)
5	70 (26.6)
6 +	12 (4.6)
Residence	
Alexandria	188 (71.5)
Bahira	26 (9.9)
Other	49 (18.6)

N: total number of respondents

Regarding to COVID-19 impact on household spending, around 63.1% of families had the same monthly income, 1.5% had an increase with a mean (SD) of 475(234.2) L.E./month, and 35.4% had a decrease with a mean (SD) of 1788.31(962.9) L.E./month. About 87.5% of families had no persons who lost their jobs due to COVID-19, 11% had only one person while 1.5% had two persons. Concerning to change of household spending, 35.4% had no change, 49.8% had increase in household spending by a mean (SD) of 1215.65(560.83) L.E./month, and 14.8% had a decrease in spending by a mean (SD) of 1702.7(989.24) L.E./month (Table 2).

**Table 2:** COVID-19 impact on household spending

Variable	N (%) N = 263	Costs (L.E.)/month			
		N*	Min	Max	Mean (SD)
Is the monthly income affected by the Corona pandemic?					
No change	166 (63.1)				
Increase	4 (1.5)	4	300	800	475(234.2)
Decrease	93 (35.4)	77	500	4000	1788.31(962.9)
How many members of your family lost their job due to the Corona pandemic?					
0	230 (87.5)				
1	29 (11.0)				
2	4 (1.5)				
How much did you spend during one month on family expenses such as food, medicine and utilities (before the outbreak of the epidemic in Egypt, March 2020)?		230	2000	9000	3769.57(1583.6)
Has there been a change in household spending during the outbreak in Egypt?					
No change	93 (35.4)				
Increase	131 (49.8)	115	500	2000	1215.65(560.83)
Decrease	39 (14.8)	37	500	4000	1702.7(989.24)

N: total number of respondents N*: total number of respondents after trimming

Concerning to COVID-19 impact on households leaving days, there is a significant observed reduction in days of leaving home before and after COVID-19 pandemic for both the respondents (before; median = 6, after; median = 5, *P* < 0.001) and for his family members (before; median = 6, after; median = 4, *P* < 0.001) (Table 3).

**Table 3:** COVID-19 impact on households leaving days

Variables	Before COVID-19 Median (Q1–Q3)	After COVID-19 Median (Q1–Q3)	Sig
How many days do you leave the house during the week?	6 (5–7)	5 (3–6)	*P* < .001
For family members, the average days that they leave the house during the week?	6 (4–7)	4 (2–6)	*P* < .001

Table 4 represents the average extra costs for each item during COVID-19 pandemic. Most of extra spending was for grocery (mean = 707.2 L.E./month, SD = 530.7), the second one was for using Taxi or Uber instead of public transportation (mean = 488.9 L.E./month, SD = 401.6), the third one was the cost of treatment for COVID-19 patients (mean = 348.4 L.E./month, SD = 344.5), and the fourth one was for periodic examination (mean = 338.3 L.E./month, SD = 160.9).

**Table 4:** Average extra costs of each item during COVID-19 pandemic

Items	N (%) N = 263	Cost (L.E.)/month			
		N*	Min	Max	Mean (SD)
Do you use a mask?					
Yes	251 (95.3)	219	50	350	157.7 (70.9)
No	12 (4.6)				
Do you buy face shield for kids?					
Yes	98 (37.3)	85	6.67	66.67	23.7 (12.3)
No	66 (25.1)				
Not applicable	99 (37.6)				
Has any of your family members been infected with COVID-19?					
Yes	69 (26.2)	60	16.67	2083.3	348.4 (344.5)
No	194 (73.8)				
Have you spent an additional cost on hand washing soap since mid-March 2020?					
Yes	141 (53.6)	114	50	500	184.3 (132.7)
No	122 (46.4)				
Has the additional cost been spent on surface disinfectants (chlorine) since mid-March 2020?					
Yes	185 (70.3)	148	30	300	110.41 (78.1)
No	78 (29.7)				
Do you use hand sanitizers?					
Yes	247 (93.9)	197	15	400	72.7 (62.1)
No	16 (6.1)				
Do you or any family member take medicines (vitamins) to prevent the emerging corona virus?					
Yes	141 (53.6)	113	40	400	135.9 (88.1)
No	122 (46.4)				
Have you been using a mouth gargle since mid-March 2020 to prevent the emerging corona virus?					
Yes	26 (9.9)	24	20	110	56.6 (27.9)
No	237 (90.1)				
Have you or any of your family members been subjected to a periodic examination or laboratory analysis since mid-March 2020 to ensure that there is no infection?					
Yes	33 (12.5)	28	100	600	338.3 (160.9)
No	230 (87.5)				
Because of the corona case, do you use a Taxi or Uber instead of public transport, in order to avoid being in crowded places?					
Yes	116 (44.1)	104	75	1275	488.9 (401.6)
No	147 (55.9)				
Were home delivery services used many times (before–after) the epidemic spread in Egypt?					
Yes–Yes	81 (30.8)				
Yes–No	23 (8.7)	23	− 300	− 300	− 300
No–No	105 (39.9)				
No–Yes	54 (20.5)	54	300	300	300
Did you increase grocery spending after the outbreak of the epidemic in Egypt?					
Yes	99 (37.6)	93	100	2000	707.2 (530.7)
No	164 (62.4)				
School expenses changes? **					
Increase	85 (62.04)	75	83.33	833.33	295.4 (199.8)
No increase	52 (37.9)				
University expenses changes?***					
Increase	30 (44.7)	26	83.3	833.3	192.3 (205.2)
No increase	37 (55.2)				

N: total number of respondents N*: total number of respondents after trimming ** families have children at school *** families have students at university

**Fig. 1:**
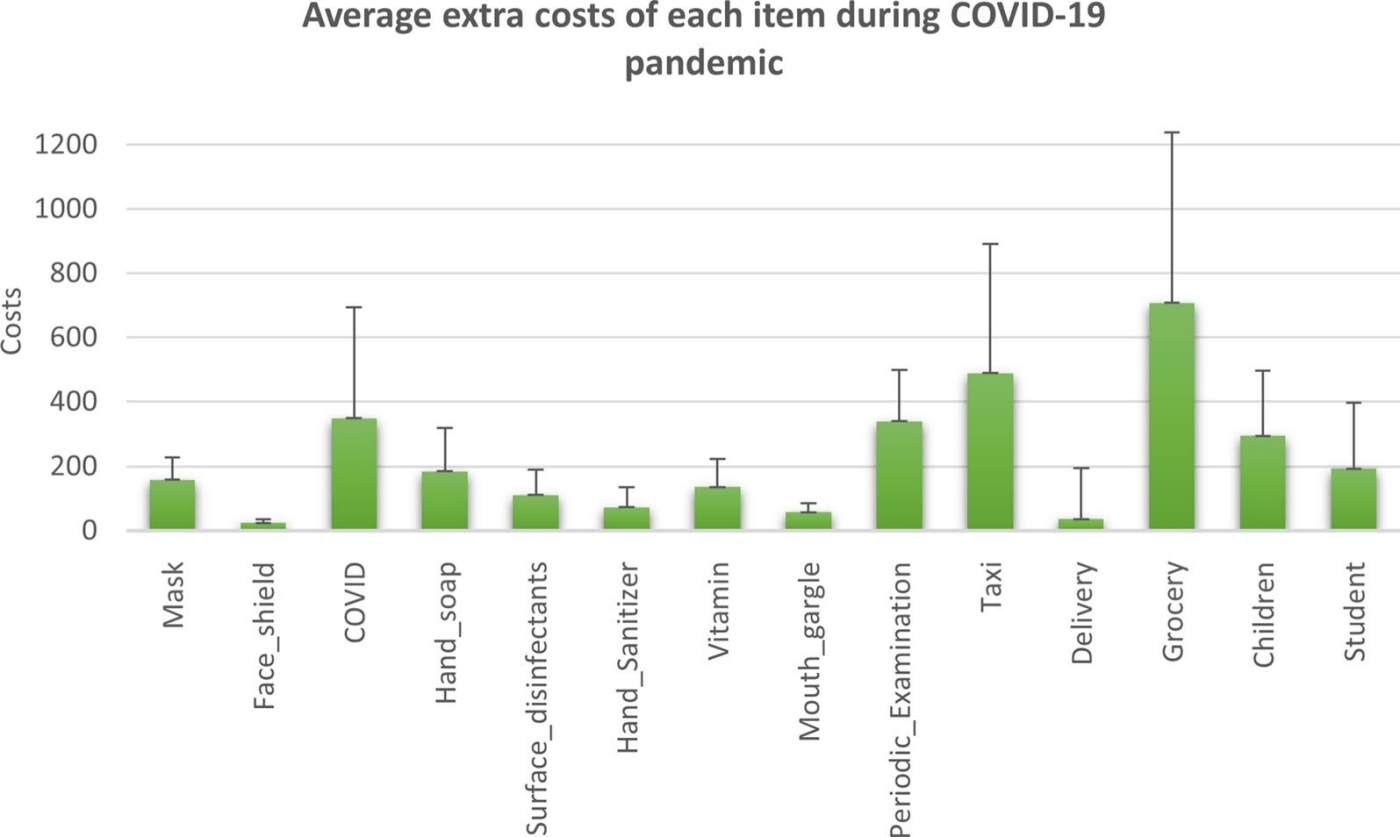
Average extra costs of each item during COVID-19 pandemic

Figure 1 shows the mean extra costs of each item during COVID-19 with SD as error bars.

**Fig. 2:**
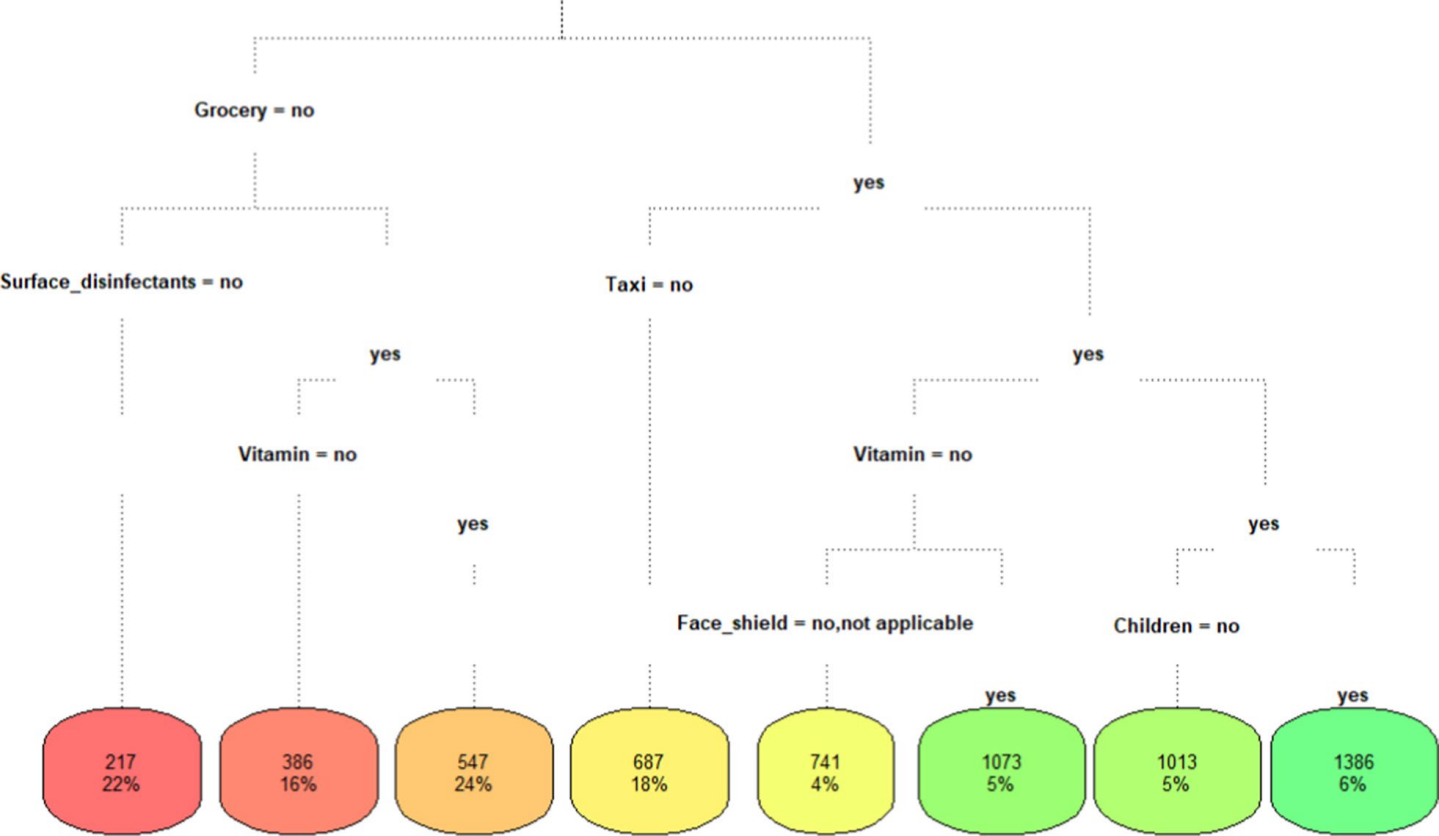
Regression tree modelling for predicting the total average extra costs per month

The regression tree selected only six items from the 14 items to build the tree. These items are grocery, surface disinfectants (chlorine), taxi, vitamin, face shield, and children. The tree has 8 leaves with a main root which is grocery item (Fig. 2).

Table 5 shows the decision rules of the regression tree, it can be read as follows; for example, if you have no increase in grocery spending and have no increase in surface disinfectants spending then the total average extra cost of spending per month is 217 L.E. Thus, the minimum average extra cost spending per month is 217 L.E. and the maximum average extra cost spending per month is 1386 L.E.

**Table 5:** Decision rules of the regression tree

Rules	Cost (L.E.)/month
When grocery is no and surface disinfectants is no	217
When grocery is no and vitamin is no and surface disinfectants is yes	386
When grocery is no and vitamin is yes and surface disinfectants is yes	547
When grocery is yes and taxi is no	687
When grocery is yes and vitamin is no and taxi is yes and face shield is no or not applicable	741
When grocery is yes and vitamin is yes and taxi is yes and children is no	1013
When grocery is yes and vitamin is no and taxi is yes and face shield is yes	1073
When grocery is yes and vitamin is yes and taxi is yes and children is yes	1386

## Discussion

During COVID-19 period, a change in household spending has occurred to prevent the pandemic of COVID-19, for example: buying extra products such as masks, hand sanitizers, vitamins or changing the routine of life such as using taxi instead of public transportation or buying more food due to the long period of staying at home. Therefore, the aim of the current study is to assess the average extra costs of each item to predict the total average extra costs in household spending.

The present study reveals that 11% of families had one person lost his job. On the other hand, a survey conducted by Adams-Prassl et al. in late March 2020 showed that 8% of workers had lost their job (Adams-Prassl et al. 2020) and another study found that 3% of employees had lost their jobs (Gardiner and Slaughter 2020). Regarding income, 62% of households reported reduction in total income (Sánchez-Páramo and Narayan 2020) compared to 35.4% in the current study this difference may be due to that our respondents have high level of education (most of them are working in medical field) and those with lower levels of education are more likely to lose their jobs (Sánchez-Páramo and Narayan 2020).

Regarding to the change of household spending due to COVID-19, 49.8% of households had increase in spending (mean = 1215.65 L.E./month, SD = 560.83) while 14.8% had decrease in spending (mean = 1702.7 L.E./month, SD = 989.24). The reason for this decrease can clarify as that many activities had been stopped or delayed due to COVID-19 pandemic.

From Income, Expenditure and Consumption survey, the average annual expenditure of the family on food and drink in Egypt is 37.1%, 33.9% in urban, and 40.2% in rural of the total annual expenditure, which is the largest percentage of the family’s expenditure (Central Agency for Public Mobilization and Statistics 2019). The present study has similar results, the increasing in grocery spending was the dominated item during COVID-19 pandemic, has the maximum average cost change (mean = 707.2 L.E./month, SD = 530.7) compared to other items which can be understand that with more time spent at home, more food we eat which led to spend more money. Moreover, physicians stated that more people reported unexpected weight gain during the COVID-19 pandemic (Sweet 2020).

Concerning regression tree, the minimum average extra cost in household spending per month was 217 L.E., while the maximum average extra cost was 1386 L.E., this result can be validated by comparing it with the change in household spending per month (min = 500 L.E., max = 2000 L.E.).

### Limitation

Due to COVID-19 pandemic, face-to-face data collection was difficult to do, and therefore, snowball sampling technique was implemented to recruit individuals, highly educated only. Thus, this result can be generalized only among highly educated people.

### Recommendation

A phone survey is needed to be conducted to recruit all different groups in the community.

### Conclusion

The effect of COVID-19 pandemic in households spending varies largely between households, it depends on what they do to prevent COVID-19.

## Data Availability

All data are available from the corresponding author on responsible request.

## Abbreviation

COVID-19: Coronavirus disease 2019
